# Supplementary material for: Crotoxin Modulates Macrophage Phenotypic Reprogramming
Source: Toxins (Basel). 2023 Oct 17;15(10):616. doi: 10.3390/toxins15100616 (PMC10611389; doi:10.3390/toxins15100616)
Supplement: Supplementary file 1 [file toxins-15-00616-s001.zip › toxins-2570252-SM-1.pdf]

No tumor group								
Body weight (g)								
(PBS+Saline)			(PBS+CTX 0.9 µg)			(PBS+CTX 5 µg)		
	Weight (1st day	Weight (13th day)		Weight (1st day)	Weight (13th day)		Weight (1st day)	Weight (13th day)
**Animal 1**	29.12	29.12	**Animal 1**	27.79	26.65	**Animal 1**	23.97	23.73
**Animal 2**	26.67	26.62	**Animal 2**	26.98	28.19	**Animal 2**	25.77	26.61
**Animal 3**	28.66	28.89	**Animal 3**	25.57	27.21	**Animal 3**	23.78	24.49
**Animal 4**	24.28	26.85	**Animal 4**	24.29	27.55	**Animal 4**	23.73	24.28
**Animal 5**	24.36	28.64	**Animal 5**	23.96	27.62	**Animal 5**	24.27	25.13
**Animal 6**	24.85	27.67	**Animal 6**			**Animal 6**	24.71	24.74

Tumor group					
Body weight (g)					
(Tumor+Saline)			(Tumor+CTX 0.9 µg)		
	Weight (1st day)	Weight (13th day)		Weight (1st day)	Weight (13th day)
**Animal 1**	28.21	36.8	**Animal 1**	29.54	34.21
**Animal 2**	26.75	35.49	**Animal 2**	29.00	34.44
**Animal 3**	28.91	36.55	**Animal 3**	26.49	33.59
**Animal 4**	24.27	31.49	**Animal 4**	26.13	31.63
**Animal 5**	25.06	34.52	**Animal 5**	25.65	33.64
**Animal 6**	24.75	32.28	**Animal 6**	27.66	38.99
**Animal 7**	25.95	36.11	**Animal 7**	27.12	37.08
**Animal 8**	26.03	35.16	**Animal 8**	27.37	34.80

	(Tumor+CTX 5 μg)	
	**Weight (1st day)**	**Weight (13th day)**
**Animal 1**	28.6	36.63
**Animal 2**	28.19	36.79
**Animal 3**	28.23	36.69
**Animal 4**	25.51	36.46
**Animal 5**	25.4	34.09
**Animal 6**	26.81	37.66
**Animal 7**	25.49	37.19
**Animal 8**	26.53	35.79
