# Supplementary material for: Crotoxin Modulates Macrophage Phenotypic Reprogramming
Source: Toxins (Basel). 2023 Oct 17;15(10):616. doi: 10.3390/toxins15100616 (PMC10611389; doi:10.3390/toxins15100616)
Supplement: Supplementary file 1 [file toxins-15-00616-s001.zip › toxins-2570252-SM-2.pdf]

Crotoxin Modulates Macrophage Phenotypic Reprogramming

Camila Lima Neves [1], Christiano Marcello Vaz Barbosa [2], Priscila Andrade Ranéia-Silva [3], Eliana L. Faquim-Mauro [4,5] and Sandra Coccuzzo Sampaio [1,6,*]

[1] Laboratory of Pathophysiology, Butantan Institute, São Paulo 05503-900, Brazil; camila.neves@butantan.gov.br
[2] Department of Biochemistry, Federal University of São Paulo, São Paulo 04044-020, Brazil; cmvbarbosa@unifesp.br
[3] University of Ribeirão Preto, Campus Guarujá, São Paulo 11440-003, Brazil; parsilva@unaerp.br
[4] Laboratory of Immunopathology, Butantan Institute, São Paulo 05503-900, Brazil; eliana.faquim@butantan.gov.br
[5] Department of Immunology, Institute of Biomedical Sciences, University of São Paulo, São Paulo, 05508-220, Brazil;
[6] Department of Pharmacology, Institute of Biomedical Sciences, University of São Paulo, São Paulo 05508-220, Brazil;
* Correspondence: sandra.coccuzzo@butantan.gov.br

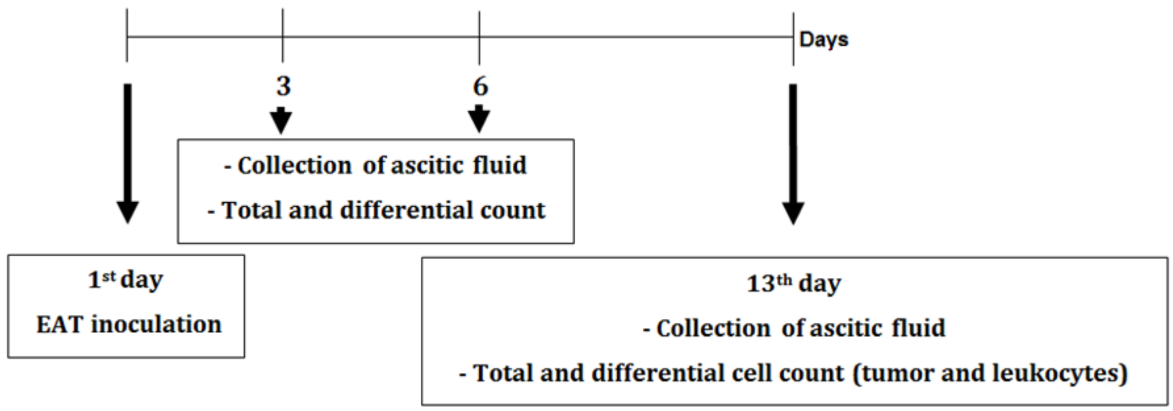

Scheme S1. Representative experimental scheme of the growth curve of the EAT.

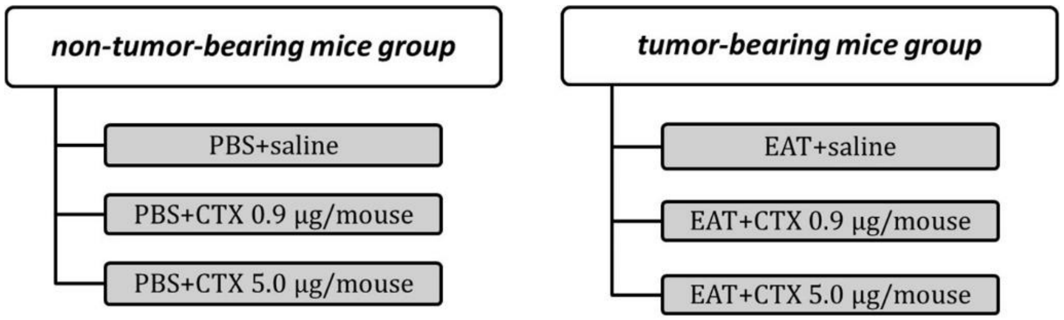

Schema S2: Representative scheme of the groups used in the experimental protocols.

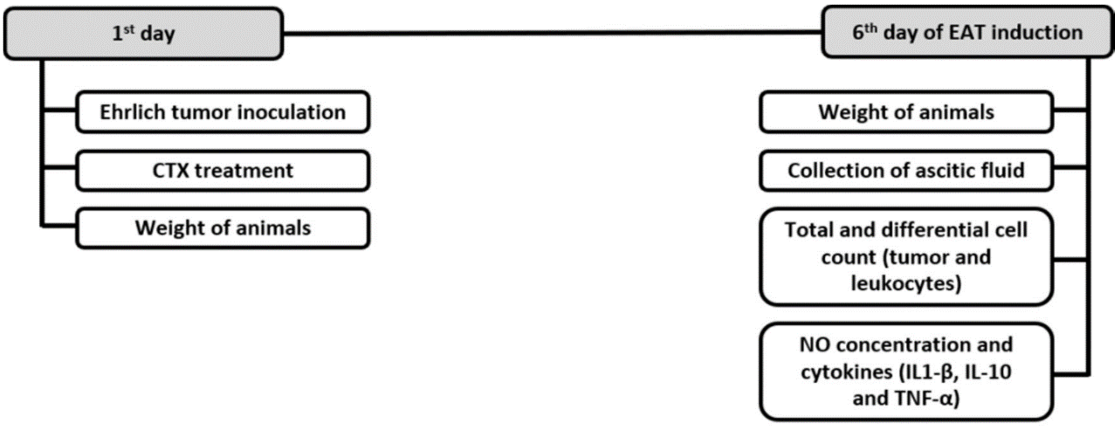

Schema S3: Representative scheme of the evaluation of different parameters on the 6th day of ascites development.

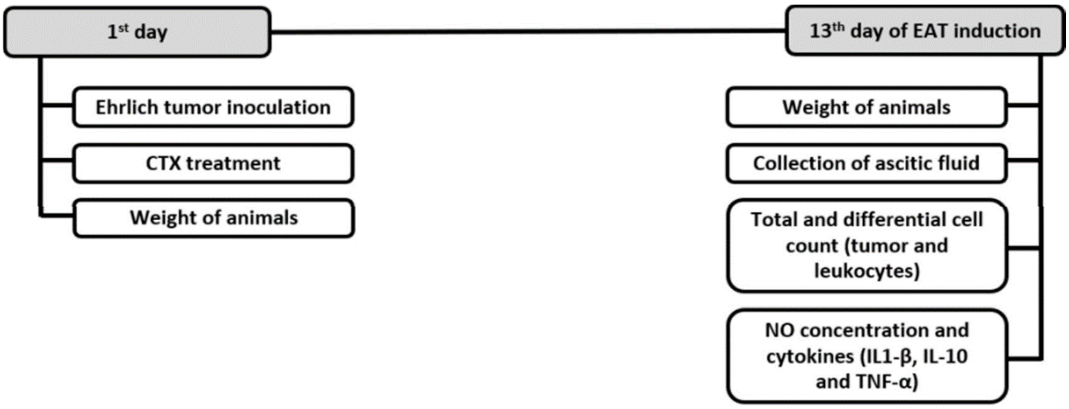

Schema S4: Representative scheme of the evaluation of different parameters on the 13th day of ascites development.
